# Supplementary material for: Hurricane María drives increased indoor proliferation of filamentous fungi in San Juan, Puerto Rico: a two-year culture-based approach
Source: PeerJ. 2022 Mar 3;10:e12730. doi: 10.7717/peerj.12730 (PMC8898552; doi:10.7717/peerj.12730)
Supplement: Supplemental Information 1 — Pairwise comparisons between the degree of water damage categories (Dry, Water-Damage, Flooded) were tested using Kruskal-Wallis to compute alpha diversity measurement (Chao 1) p-values. [file peerj-10-12730-s001.docx]

| Group1 | Group2 | Group1 mean | Group1 std | Group2 mean | Group2 std | t stat | p-value |
| --- | --- | --- | --- | --- | --- | --- | --- |
| Flooded | Water-Damage | 3.11052632 | 1.34206146 | 3.04868687 | 1.28376029 | 0.37897571 | 1 |
| Flooded | Dry | 3.11052632 | 1.34206146 | 2.85381526 | 1.1162105 | 1.36806103 | 0.576 |
| Dry | Water-Damage | 2.85381526 | 1.1162105 | 3.04868687 | 1.28376029 | -1.2008103 | 0.69 |
