# Supplementary material for: Hurricane María drives increased indoor proliferation of filamentous fungi in San Juan, Puerto Rico: a two-year culture-based approach
Source: PeerJ. 2022 Mar 3;10:e12730. doi: 10.7717/peerj.12730 (PMC8898552; doi:10.7717/peerj.12730)
Supplement: Supplemental Information 2 — Pairwise comparisons between the sampling site (Indoor, Outdoor) and sampling year (First, Second) categories were tested using Kruskal-Wallis to compute alpha diversity measurement (Chao 1) p-values. [file peerj-10-12730-s002.docx]

| Group1 | Group2 | Group1 mean | Group1 std | Group2 mean | Group2 std | t stat | p-value |
| --- | --- | --- | --- | --- | --- | --- | --- |
| IndoorSecond | IndoorFirst | 2.90390625 | 1.2513352 | 2.82564103 | 1.03916089 | 0.3836915 | 1 |
| IndoorSecond | OutdoorFirst | 2.90390625 | 1.2513352 | 2.62222222 | 0.86493773 | 0.88569618 | 1 |
| IndoorFirst | OutdoorFirst | 2.82564103 | 1.03916089 | 2.62222222 | 0.86493773 | 0.75151404 | 1 |
| IndoorFirst | OutdoorSecond | 2.82564103 | 1.03916089 | 3.41538462 | 1.02232477 | -1.8487889 | 0.414 |
| OutdoorFirst | OutdoorSecond | 2.62222222 | 0.86493773 | 3.41538462 | 1.02232477 | -2.2562136 | 0.18 |
| IndoorSecond | OutdoorSecond | 2.90390625 | 1.2513352 | 3.41538462 | 1.02232477 | -1.364903 | 1 |
